# A high-throughput screen for nucleolar function reveals a role for the signaling protein, SPRR3, in ribosome biogenesis

**DOI:** 10.1016/j.jbc.2026.111132

**Published:** 2026-01-07

**Authors:** Emily C. Sutton, Carson J. Bryant, Janina I.S. Gbenoba, Isabella R. Lawrence, Susan J. Baserga

**Affiliations:** 1 Department of Molecular Biophysics and Biochemistry, Yale University and the Yale School of Medicine, New Haven, Connecticut, USA; 2 Department of Therapeutic Radiology, Yale School of Medicine, New Haven, Connecticut, USA; 3 Department of Genetics, Yale School of Medicine, New Haven, Connecticut, USA

**Keywords:** Ribosome biogenesis, nucleolus, RNA polymerase I, pre-rRNA, nucleolar stress, TP53

## Abstract

SPRR3 is a small, proline-rich protein that promotes cell proliferation. Overexpressed SPRR3 is associated with cancer and regulates AKT phosphorylation at serine 473. However, the specific cellular mechanisms by which SPRR3 drives proliferation are not fully understood. Using a genome-wide siRNA screen in MCF10A breast epithelial cells for decreased nucleolar number, we identified SPRR3 as a novel regulator of ribosome biogenesis. We used siRNA to deplete SPRR3 and found that it is required for transcription of the pre-ribosomal RNA (pre-rRNA), the earliest step in ribosome biogenesis. Furthermore, this reduction in pre-rRNA transcription triggers the nucleolar stress response (increased TP53 protein and *CDKN1A* mRNA levels) in both MCF10A cells and A549 lung carcinoma cells. Finally, SPRR3 depletion reduces AKT phosphorylation in both cell lines and correlates with lower levels of the RNAPI catalytic subunit POLR1A. In sum, we establish a new role for the non-nucleolar protein SPRR3 in ribosome biogenesis, specifically pre-rRNA transcription, *via* its ability to facilitate phosphorylation of AKT.

The process of making ribosomes, the large ribonucleoproteins that translate mRNA into protein, occurs in the nucleolus of all eukaryotic cells. Ribosome biogenesis is a complex series of steps that involves the transcription of the primary pre-ribosomal RNA transcript (47S pre-rRNA) by RNA polymerase I (RNAPI) and its cleavage and modification to produce the mature 18S, 5.8S, and 28S rRNAs in humans (1, 2, 3, 4). As the pre-rRNAs are cleaved and modified, they are assembled with 80 ribosomal proteins to produce the mature small subunit (40S, SSU) and large subunit (60S, LSU) of the ribosome. The nucleolar steps of ribosome biogenesis that lead to the production of mature ribosomes involve many protein factors as well as small nucleolar ribonucleoproteins (snoRNPs). While we now know the identity and function of the many nucleolar components that contribute to making mature ribosomes, the regulation of ribosome biogenesis by non-nucleolar proteins and processes is less well-understood.

To identify proteins required for making ribosomes in human cells using an unbiased approach, we previously established a phenotypic cell-based assay for nucleolar number in MCF10A breast epithelial cells (5, 6, 7). The assay measures nucleolar numbers in high throughput, and we have demonstrated that a change in nucleolar number accurately reports decreased nucleolar function. We applied this assay to a genome-wide siRNA screen to discover proteins required for making ribosomes. Using robust screening statistics, we identified hits for proteins localized both in and outside of the nucleolus. We have used this screen to define and explore the function of a number of nucleolar and non-nucleolar proteins in ribosome biogenesis (8, 9, 10). The small proline-rich protein 3 (SPRR3, or esophagin) is one of the hits in our genome-wide siRNA screen for nucleolar function.

SPRR3 is a 17 kDa protein that is 22% proline (35/158 amino acids; uniprot.org) (11, 12, 13). It is one member of a family of proteins implicated in the development of the cornified cell envelope of stratified squamous epithelia. Mutations in SPRR3 have been associated with atopic dermatitis and asthma (14, 15). SPRR3 is highly expressed in the oral mucosa, esophagus, gall bladder, urinary bladder, vagina, cervix, and tonsils in humans (16). Although contemporary databases show it to be expressed in skin (16), early reports suggested SPRR3 was unique in its family of proteins as the only member not detectable in human skin (11, 12). Higher SPRR3 expression drives cell proliferation and cancer in colorectal, non-small cell lung, breast, pancreatic, and glioblastoma cancers and in clear cell renal carcinoma, but downregulation of SPRR3 is seen in esophageal cancers (17, 18, 19, 20, 21, 22, 23, 24). With the exception of esophageal cancer, SPRR3 is considered a driver of proliferation and tumorigenesis, possibly due to its ability to maintain AKT phosphorylation (17, 18, 19). Interestingly, AKT phosphorylation has been shown to activate nucleolar ribosome biogenesis *via* RNAPI (25, 26), hinting at a mechanism for SPRR3’s ability to enhance cell growth.

Here, we provide evidence for a novel role for SPRR3 in nucleolar ribosome biogenesis in MCF10A breast epithelial cells and in A549 lung carcinoma cells. siRNA depletion of SPRR3 with siGENOME siRNAs in MCF10A cells causes a decrease in the average number of nucleoli per nucleus. Validation with the higher fidelity siON-TARGET (siONT) siRNA SMARTpool reproduces this finding. siRNA deconvolution, the process of testing each of the siONT RNAs individually, reveals that three-fourths of the siRNAs reduce nucleolar number and cell viability. Using a custom pool of two siONT RNAs across several different assays, we show that SPRR3 is required for RNAPI transcription of the pre-rRNA, the first step in ribosome biogenesis. As expected with decreased ribosome synthesis due to RNAPI inhibition, we observe a concomitant reduction in translation (protein synthesis) by the puromycin incorporation assay. We report that a decrease in SPRR3 levels triggers the nucleolar stress response in MCF10A cells and an even more robust response in A549 cells. Finally, we find that depletion of SPRR3 reduces levels of phosphorylated AKT (pAKT) in both MCF10A and A549 cell lines, and that levels of the essential RNAPI subunit POLR1A decrease, pointing to a potential mechanism by which SPRR3 regulates early ribosome biogenesis. Taken together, we show for the first time that SPRR3 plays a critical role in ribosome biogenesis and that phosphorylation of AKT facilitated by SPRR3 mediates this effect.

## Results

### SPRR3 is a hit on an unbiased screen for nucleolar function

Previously, we have shown that defective ribosome biogenesis resulting from siRNA depletion of ribosome biogenesis factors correlates with a change in the number of nucleoli in MCF10A breast epithelial cells (5, 6, 7). Typical MCF10A cells have 2 to 3 nucleoli per nucleus, and an increase or decrease in nucleolar number reflects defective ribosome biogenesis. We screened the human genome by siRNA depletion (siGENOME) using a high-throughput phenotypic approach, where we quantified the number of nucleoli per nucleus. In this screen, nucleoli were detected by anti-fibrillarin antibody (72B9; (27)), and nuclei were detected by Hoechst staining, followed by analysis with a CellProfiler pipeline to quantify the number of nucleoli per nucleus (6). siUTP4, a nucleolar SSU processome protein, was used as a positive control while siGFP was used as a negative control.

**Figure 1:**
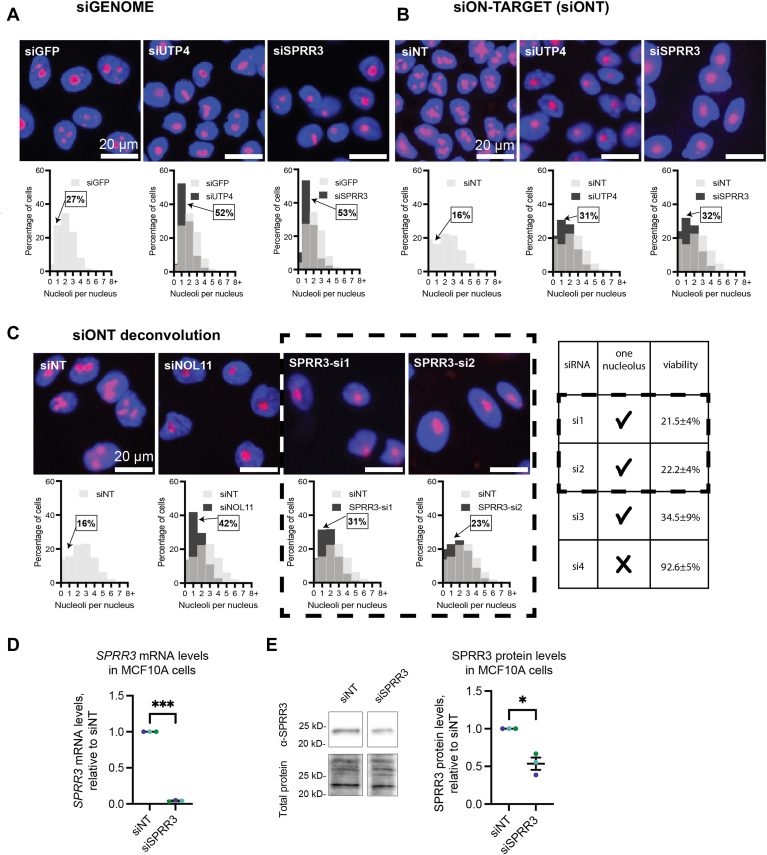
**siSPRR3 depletion results in a reduction in the number of nucleoli per nucleus in MCF10A cells.***A*, siSPRR3 depletion with siGENOME siRNAs reduces the number of nucleoli per nucleus. Images and histograms from a genome-wide screen using Dharmacon/Horizon siGENOME siRNAs (6). The images show nuclei (Hoechst, *blue*) and nucleoli (anti-fibrillarin, *red*) after 72 h of treatment with the negative control (siGFP), positive control (siUTP4), or siSPRR3 siRNAs. Histograms show the distribution of cells that have the indicated number of nucleoli per nucleus. *Light grey* shows the distribution for the negative control, *black* shows the test condition, and dark grey shows overlap of the two frequencies. *B*, siSPRR3 depletion with siON-TARGET (siONT) SMARTPool siRNA reduces the number of nucleoli per nucleus. The images show nuclei and nucleoli after 72 h of treatment with the negative control (non-targeting siRNA, siNT), positive control (siUTP4), or siSPRR3 siRNAs. The shading in the histograms is as in *A*). The data were collected across four replicates which are pooled in the histogram. *C*, siSPRR3 depletion with individual siONT SMARTPool siRNAs (deconvolution) reduces the number of nucleoli per nucleus. The images show nuclei and nucleoli after 72 h of treatment with the negative control (siNT), positive control (siNOL11), or representative siSPRR3 individual siRNAs (SPRR3-si1 and SPRR3-si2). The shading in the histograms is as in (*A*). The histograms include all three replicates pooled. The table summarizes each of four individual siONT SMARTPool siRNAs, showing that a reduction in nucleolar number correlates with a loss in cell viability. *D*, siSPRR3 (custom subpool of SPRR3-si1 and SPRR3-si2) reduces *SPRR3* mRNA levels in MCF10A cells. RT-qPCR data of *SPRR3* mRNA demonstrating knockdown after 72 h. The data were normalized to 7SL RNA abundance, then to siNT for comparison using the ΔΔC_T_ method. The mean ± SEM are shown alongside individual data points, colored by replicate. *E*, siSPRR3 (custom subpool of SPRR3-si1 and SPRR3-si2) reduces SPRR3 protein levels in MCF10A cells. Western blot of SPRR3 protein demonstrating decreased levels after 72 h. Protein levels were normalized to total protein (trichloroethanol total protein stain), then to siNT. The mean ± SEM are shown alongside individual data points, colored by replicate. This sample was run on the same Western blot as in (32). After imaging total protein on the membrane, the blot was cut between 10 to 15 kD markers to stain separately for SPRR3 (*E*, above) or RPS28 (32). Data in (*D* and *E*) were analyzed by unpaired two-sided Welch's *t*-tests in GraphPad Prism. ∗, *p* < 0.05; ∗∗∗, *p* < 0.001.

While we expected known nucleolar proteins as hits (10, 28), non-nucleolar proteins also emerged as potential nucleolar regulators, including SPRR3 (Fig. 1*A*). siRNA depletion of SPRR3 results in a higher percentage of cells with one nucleolus per nucleus compared to the negative control (siGFP), shown by cell images and their quantitation in histograms (Fig. 1*A*). While 27% of cells in the negative control (siGFP) had one nucleolus per nucleus, nearly twice that number (53% of cells) had one nucleolus after 72 h of treatment with siSPRR3, comparable to the siUTP positive control (52% of cells). We thus identified SPRR3 as a hit in the siGENOME genome-wide screen.

To validate these results using higher fidelity siRNAs, we re-screened MCF10A cells with siON-TARGET (siONT) SMARTpool siRNAs and observed similar results. siONT SPRR3 SMARTpool depletion displayed double the percentage of cells with one nucleolus (32%) compared to the negative control (siNT; 16%) as visualized in the images and histograms (Fig. 1*B*). This was again comparable to the positive control siUTP4 (31% of cells in this screen). These results are consistent with those that we obtained with the siGENOME siRNAs and confirm SPRR3 as a hit.

To minimize the likelihood that the reduced nucleolar number we observed was the result of an off-target effect, we performed siRNA pool deconvolution experiments. The siONT SMARTpool that targets siSPRR3 is composed of four individual siRNAs. If two or more of these leads to a reduction in nucleolar number, it is more likely that SPRR3 is a *bona fide* hit. We tested each of the four individual siRNAs in the siONT SMARTpool in triplicate. A non-targeting siRNA (siNT) and an siRNA that depletes the essential nucleolar protein NOL11 (siNOL11) were used as negative and positive controls, respectively. SPRR3 passed this validation, with three of the four individual siRNAs targeting SPRR3 (siSPRR3-si1, siSPRR3-si2, and siSPRR3-si3) causing a reduction in nucleolar number. Representative images and histograms are shown for siSPRR3-si1 and siSPRR3-si2 in Figure 1*C*, with a summary table for all four siRNAs. Thus, it is likely that the reduced nucleolar number we observe is due to the depletion of SPRR3.

Because ribosome biogenesis is a critical process carried out by many essential proteins (3, 4), we have previously observed that depletion of screen hits also results in reduced cell viability (10, 28, 29). As expected, we observe this for SPRR3 depletion, where reduced viability was seen for each of the siRNAs that caused an increase in the percentage of cells with one nucleolus (Fig. 1*C*; siONT si1, si2, si3). The effects of SPRR3 depletion on the nucleolus were thus deleterious to cell growth, which we hypothesize is due to reduced ribosome biogenesis.

We created a custom equimolar subpool of two individual SPRR3 siRNAs (siSPRR3-si1 and siSPRR3-si2 in Fig. 1*C*) and used it to confirm knockdown of both the *SPRR3* mRNA transcript and the SPRR3 protein. After 72 h of treatment with the custom siRNA subpool, *SPRR3* mRNA levels were greatly reduced (Fig. 1*D*), and SPRR3 protein levels were reduced to half of endogenous levels (Fig. 1*E*). This is comparable to previously published results showing depletion of SPRR3 with siRNA (18, 19). Except where otherwise noted, this custom subpool was used for all subsequently described assays in which SPRR3 was depleted with siRNA.

Based on the observations that SPRR3 depletion results in a decrease in nucleolar number with multiple siRNAs (siGENOME, siONT pool, 3/4 individual siONT siRNAs) with a concomitant decrease in cell viability, we concluded that SPRR3 is a promising candidate as a novel protein regulator of ribosome biogenesis.

### SPRR3 is a novel regulator of RNAPI transcription

**Figure 2:**
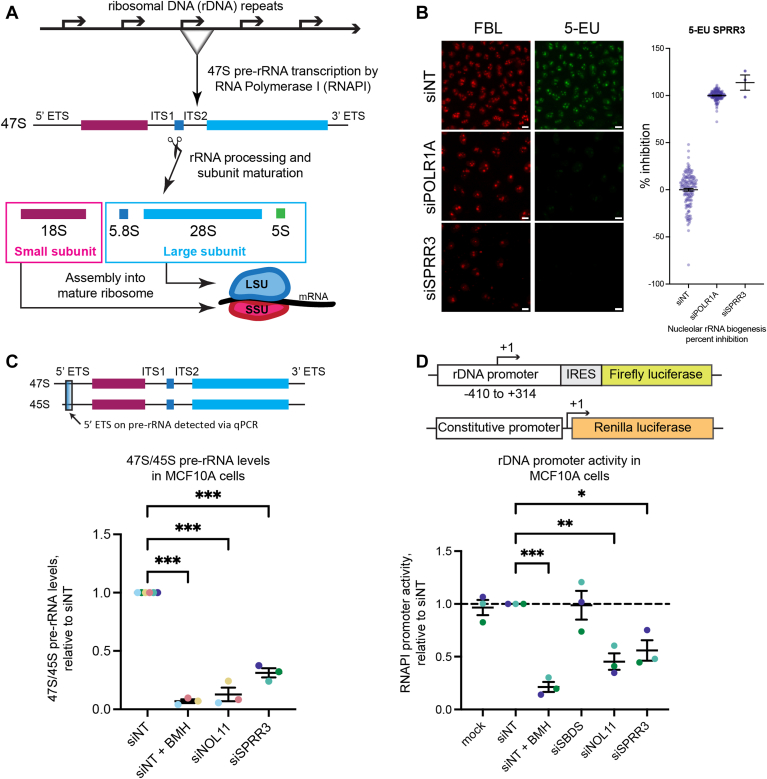
**SPRR3 depletion reduces pre-rRNA transcription.***A*, schematic of the major steps in ribosome biogenesis in human cells. *B*, SPRR3 depletion inhibits nucleolar rRNA biogenesis. Representative images and quantification of control or SPRR3-depleted MCF10A cells (siONT SMARTpool) following anti-fibrillarin (FBL) staining and 5-EU incorporation. Scale bars are 10 μm. siNT is a non-targeting negative control siRNA. siPOLR1A is a positive control targeting the RNAPI subunit, POLR1A. The overall mean percent inhibition ± SEM is shown for each treatment, with each dot representing one well. Each well in the siSPRR3 condition represents a separate day of testing and control datapoints are distributed across the three testing days. 0% inhibition is determined by the mean value of siNT, and 100% inhibition is set to the mean value of the siPOLR1A positive control. *C*, RT-qPCR analysis shows decreased 47S/45S pre-rRNA levels upon SPRR3 depletion in MCF10A cells. The mean ± SEM are shown alongside individual data points, colored by replicate. The data were normalized to 7SL RNA abundance, then to siNT for comparison using the ΔΔC_T_ method. *D*, dual-luciferase reporter assay shows RNAPI promoter activity is reduced after SPRR3 depletion. The mean ± SEM are shown alongside individual data points, colored by replicate. The controls in these data have also been published in (32), without the inclusion of siSPRR3. All the data in this figure were analyzed by ordinary one-way ANOVA with multiple comparisons against siNT and Holm-Šídák correction in GraphPad Prism. ∗, *p* < 0.05; ∗∗, *p* < 0.01; ∗∗∗, *p* < 0.001.

We wanted to determine which stages of ribosome biogenesis require SPRR3. Ribosome biogenesis involves the transcription of a primary rRNA transcript, the processing of that transcript into mature rRNAs, and assembly of rRNA with ribosomal proteins to form mature ribosomes that carry out translation (Fig. 2*A*). We conducted a series of assays to measure the effects of SPRR3 depletion on the various steps of this process. For the earliest step of pre-rRNA transcription, we performed a 5-ethynyluridine (5-EU) incorporation assay, RT-qPCR to measure 47S/45S pre-rRNA transcript levels and an rDNA promoter activity reporter assay.

The 5-EU incorporation assay, previously described in detail by Bryant and McCool *et al.* (30), utilizes the clickable uridine analog 5-EU, which is incorporated into newly synthesized RNA. After treatment with 5-EU, we fix and permeabilize cells, then attach a fluorophore to 5-EU using click chemistry for imaging with microscopy. A decrease in 5-EU incorporation indicates that RNA synthesis is reduced. We measure nucleolar rRNA biogenesis specifically by quantifying only the 5-EU that colocalizes with the nucleolus, which is demarcated by anti-fibrillarin staining.

We performed this assay after SPRR3 depletion with the siONT SMARTpool and compared it to our two controls, the negative control siNT and the positive control siPOLR1A, which depletes the major catalytic subunit of RNAPI (Fig. 2*B*). We calculated a percentage inhibition of nucleolar signal by setting siNT to 0% inhibition and siPOLR1A to 100% inhibition. siSPRR3 percentage inhibition was on average 114%, comparable to the siPOLR1A positive control, leading us to conclude that SPRR3 is required for nucleolar rRNA biogenesis.

We measured levels of the primary pre-rRNA transcript using RT-qPCR as previously described (9, 29, 31, 32). We used primers that span the 5′ ETS region of the 47S/45S pre-rRNAs, which is cleaved off very early in pre-rRNA processing (Fig. 2*C*). RT-qPCR targeting of this region is therefore a suitable proxy for primary pre-rRNA transcript levels. Depletion of SPRR3 caused a significant decrease in levels of this 47S/45S transcript to 31% of negative control levels (siNT), approaching the levels obtained with the positive controls, including treatment with RNAPI inhibitor BMH-21 (33, 34) and siNOL11 (Fig. 2*D*). This experiment supports the conclusion that depletion of SPRR3 leads to reduced pre-rRNA transcription.

We performed a reporter gene assay that has been used previously to measure rDNA promoter activity (7, 10, 32, 35) (Fig. 2*D*). In this system, the human rDNA promoter drives Firefly luciferase bearing an internal ribosome entry site (IRES). A plasmid constitutively expressing the *Renilla* luciferase protein is co-transfected to normalize for transfection efficiency. Activity of the rDNA promoter was reduced to 56% upon SPRR3 depletion, a change similar to that caused by the positive siRNA control, siNOL11 (45%) (Fig. 2*D*). RNAPI inhibitor BMH-21 was also used as a positive control (21%) (Fig. 2*D*). Negative controls were mock treatment (no siRNA), siNT and siSBDS. SBDS, Shwachman-Bodian-Diamond Syndrome Protein, is a protein that affects late ribosome biogenesis/assembly, but not RNAPI transcription. This is additional experimental evidence that depletion of SPRR3 leads to reduced pre-rRNA transcription through reduced RNAPI activity at the rDNA promoter.

We also measured the extent to which SPRR3 depletion affects the pre-rRNA processing steps that occur after transcription. The ratio of mature 28S to 18S rRNA can be calculated using the Agilent Bioanalyzer, and changes in this ratio indicate reduced levels of either the large or small ribosomal subunit, respectively (9, 31, 32). No change in ratio was observed upon SPRR3 depletion (Fig. S1). Lastly, no changes were seen when we completed northern blots to assess levels of selected pre-rRNA intermediates (Fig. S2). Combined, these results suggest that SPRR3 is not required for pre-rRNA processing. Together with the previously described assays, we conclude that SPRR3 primarily plays a role in the earliest step of ribosome biogenesis: pre-rRNA transcription.

### SPRR3 is required for optimal protein synthesis

**Figure 3:**
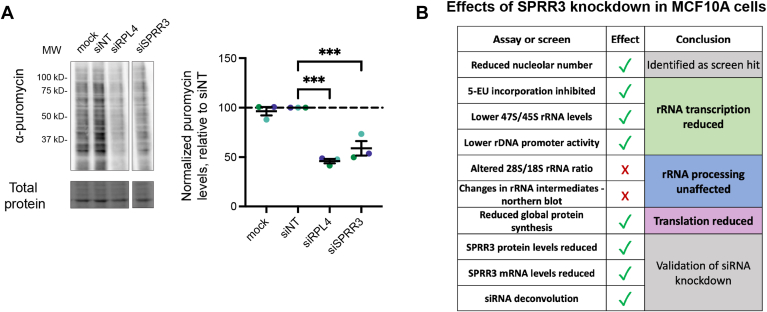
**SPRR3 depletion causes reduced global translation (protein synthesis).***A*, representative image and quantification of puromycin incorporation shows reduced translation after SPRR3 depletion in MCF10A cells. α-puromycin shows puromycin incorporation as a proxy for global protein synthesis. Total protein is the trichloroethanol total protein stain loading control. Images were quantified with Bio-Rad Image Lab. The mean ± SEM are shown alongside individual data points, colored by replicate. siRPL4 is the positive control. Data were normalized to a non-targeting siRNA (siNT), then graphed and analyzed by ordinary one-way ANOVA with multiple comparisons against siNT and Holm-Šídák correction in GraphPad Prism. ∗∗∗, *p* < 0.001. The control data in this puromycin Western blot have also been published in (32) without the SPRR3 data. *B*, summary table describing the effects of SPRR3 depletion on ribosome biogenesis and the nucleolus. SPRR3 depletion reduces nucleolar number, nucleolar rRNA biogenesis (5-EU incorporation assay), pre-rRNA transcript levels (RT-qPCR of 47S/45S rRNA), rDNA promoter activity (luciferase reporter assay), and global protein synthesis (puromycin incorporation assay). SPRR3 depletion was found to have no effect on the ratio of 18S to 28S rRNA nor to produce changes in pre-rRNA northern blots, indicating that SPRR3 does not affect rRNA processing. Taken together, this suggests that SPRR3 plays a role in pre-rRNA transcription and nucleolar rRNA biogenesis that is essential to the normal translational activity of ribosomes.

We have established that depletion of SPRR3 causes a reduction in ribosome biogenesis, specifically the earliest step of pre-rRNA transcription. Because of reduced pre-rRNA transcription, we would expect reduced translation, as this is the function of ribosomes. To examine this, we conducted a puromycin incorporation assay to measure active global translation, which we have done previously (6, 8, 10, 29, 31, 32). We added puromycin to cells for 1 h before protein extraction to allow its incorporation into nascent polypeptides. We then extracted proteins and performed Western blotting with an anti-puromycin antibody to detect the level of active translation among samples. For positive control, we depleted the large ribosomal subunit protein RPL4 (siRPL4). Imaging and quantification showed a significant decrease in puromycin incorporation to 59% after SPRR3 depletion (Fig. 3*A*), approaching levels obtained with the positive control siRPL4. siNT and mock treatment (no siRNA) were used as negative controls.

Altogether, we comprehensively examined the effect of SPRR3 depletion on nucleolar number, cell viability, early and late steps of ribosome biogenesis, and global translation (summarized in Fig. 3*B*) in the MCF10A cell line. We conclude that SPRR3 is required for optimal ribosome biogenesis in MCF10A cells, specifically for the transcription of pre-rRNA, the first step of ribosome biogenesis.

### SPRR3 depletion triggers the nucleolar stress response

**Figure 4:**
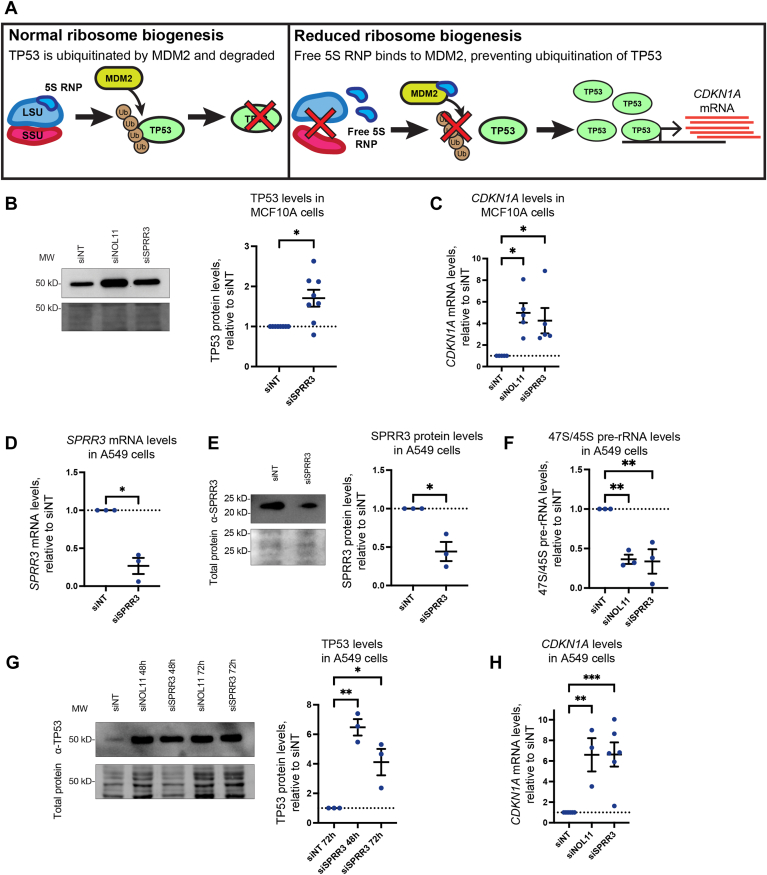
**SPRR3 depletion causes the nucleolar stress response in MCF10A and A549 cells.***A*, schematic of the nucleolar stress response in human cells. Disruption of ribosome biogenesis causes accumulation of free 5S RNP which inhibits the ubiquitin ligase MDM2, leading to accumulation of TP53 and increased transcription of *CDKN1A*. *B*, TP53 stabilization after SPRR3 depletion in MCF10A cells. Representative images and quantification of TP53 Western blots after 72h of treatment with siSPRR3. siNOL11 was used as a positive control. TP53 levels were normalized to total protein (trichloroethanol stain), then siNT. *C*, *CDKN1A* mRNA levels are elevated after SPRR3 depletion in MCF10A cells. After 72 h of siSPRR3 treatment, *CDKN1A* mRNA transcript levels were detected with RT-qPCR using primers for *CDKN1A* mRNA. siNOL11 was used as a positive control. These data were normalized to 7SL RNA abundance, then to siNT for comparison using the ΔΔC_T_ method. *D*, siSPRR3 reduces *SPRR3* mRNA levels in A549 cells. After 72 h of siSPRR3 treatment, *SPRR3* transcript levels were detected by RT-qPCR. The data were normalized as in (*C*). *E*, siSPRR3 reduces SPRR3 protein levels in A549 cells. After 72 h of treatment with siSPRR3, SPRR3 protein levels were detected by Western blot. SPRR3 levels were normalized as in (*B*). *F*, SPRR3 depletion lowers 47S/45S pre-rRNA levels in A549 cells. Primers to the 5′ ETS of the 47S/45S rRNA were used to detect pre-rRNA levels after 72h of treatment with siSPRR3. siNOL11 was used as a positive control. The data were normalized as in (*C* and *D*). *G*, TP53 stabilization after SPRR3 knockdown in A549 cells. Representative images and quantification of TP53 Western blots after 48h or 72h of treatment with siSPRR3 are shown. Protein levels were normalized as in (*B* and *E*). *H*, *CDKN1A* levels are elevated after SPRR3 depletion in A549 cells. After 72 h of treatment with siSPRR3, *CDKN1A* transcript levels were detected with RT-qPCR using primers for *CDKN1A* mRNA. siNOL11 was used as a positive control. Data were normalized as in (*C*). The mean ± SEM are shown alongside individual data points. For graphs with two conditions, data were analyzed by unpaired two-sided Welch's *t*-tests in GraphPad Prism. For graphs with more than two conditions, data were analyzed by ordinary one-way ANOVA with multiple comparisons against siNT and Holm-Šídák correction in GraphPad Prism. ∗, *p* < 0.05; ∗∗, *p* < 0.01; ∗∗∗, *p* < 0.001.

We sought to determine whether depletion of SPRR3 triggers the nucleolar stress response. Under normal conditions, the protein MDM2 ubiquitinates the tumor suppressor protein TP53, which targets it for degradation and keeps its endogenous levels sufficiently low as to not trigger an apoptotic response or cell cycle arrest (Fig. 4*A*). When ribosome biogenesis is reduced, including when RNAPI transcription is reduced, excess levels of the 5S RNP (comprised of the 5S rRNA, RPL11/uL5, and RPL5/uL18) accumulate in the cell (Fig. 4*A*). This excess 5S RNP binds to MDM2 and prevents it from ubiquitinating TP53, causing TP53 levels to increase. Increased levels of TP53 promote transcription of pro-apoptotic factors such as *CDKN1A* (aka p21). This process, known as the nucleolar stress response, is important for regulating cell proliferation and has been investigated as a potential target for anti-cancer therapies (36, 37, 38, 39).

We hypothesized that reduced pre-rRNA transcription caused by SPRR3 depletion would induce the nucleolar stress response. To examine this, we measured TP53 protein levels and *CDKN1A* mRNA levels after 72 h of SPRR3 depletion in MCF10A cells, with negative (siNT) and positive (siNOL11) controls. We performed this across eight replicates. A mean increase of 1.7-fold in TP53 protein levels was observed after 72 h of siRNA treatment with siSPRR3 (Fig. 4*B*), although this increase was highly variable. Despite this variability, a mean increase of 4.3-fold was seen in *CDKN1A* mRNA levels under the same conditions (Fig. 4*C*). We conclude that the TP53 increase is sufficient to trigger transcriptional reprogramming associated with the nucleolar stress response.

Due to the variable TP53 response in MCF10A cells, we pursued confirmation of the nucleolar stress response after SPPR3 depletion in a different cell line. We selected A549 lung carcinoma cells due to their established use in the study of nucleolar stress (40, 41, 42, 43, 44) and previous use to study the effects of SPRR3 depletion (18). We confirmed that siSPRR3 led to reduced *SPRR3* mRNA levels (to 27%) and protein levels (to 44%) in A549 cells (Fig. 4, *D* and *E*). This decrease was consistent with our observations in MCF10A cells (Fig. 1, *D* and *E*) and previous reports (18, 19). We confirmed that SPRR3 is required for pre-rRNA transcription in A549 cells by examining 47S/45S pre-rRNA levels *via* RT-qPCR after SPRR3 depletion (Fig. 4*F*). We confirmed that pre-rRNA transcription is reduced to 34% of negative control levels, comparable to the 31% that we observed in MCF10A cells (Fig. 2*C*) and the positive control in A549 cells (siNOL11, 36%).

After confirming that SPRR3 depletion reduces pre-rRNA transcription in A549 cells, we measured levels of TP53 in A549 cells upon siSPRR3 treatment. We found that TP53 levels were increased 6.5-fold after 48 h, and 4.1-fold after 72 h. *CDKN1A* levels were subsequently elevated 6.6-fold at 72 h (Fig. 4*H*). We conclude that SPRR3 depletion triggers the nucleolar stress response in both cell lines, with a response of higher magnitude observed in A549 cells, which are well-established for the study of the nucleolar stress response. Taken together, these results connect SPRR3 to pre-rRNA transcription and to cell growth *via* the nucleolar stress response.

### SPRR3 depletion reduces AKT phosphorylation

Since SPRR3 is not a known nucleolar protein, we reasoned that SPRR3’s established role in cell growth and signaling might provide an underlying mechanism for its requirement in pre-rRNA transcription. Previous work has found that SPRR3 regulates the PI3K/AKT pathway and phosphorylation of AKT at serine 473 (pAKT) (18, 19). Furthermore, AKT phosphorylation at this site is associated with active RNAPI transcription and cell proliferation (25, 26). siRNA depletion of SPRR3 has been shown to reduce pAKT relative to AKT (19), including in A549 cells (18). Notably, reduced AKT phosphorylation can reduce RNAPI transcription of rRNA, both directly and as a result of subsequent mTORC1 activation (26). We therefore hypothesized that reduced phosphorylation of AKT upon SPRR3 depletion may drive the reduced pre-rRNA transcription that we observe.

**Figure 5:**
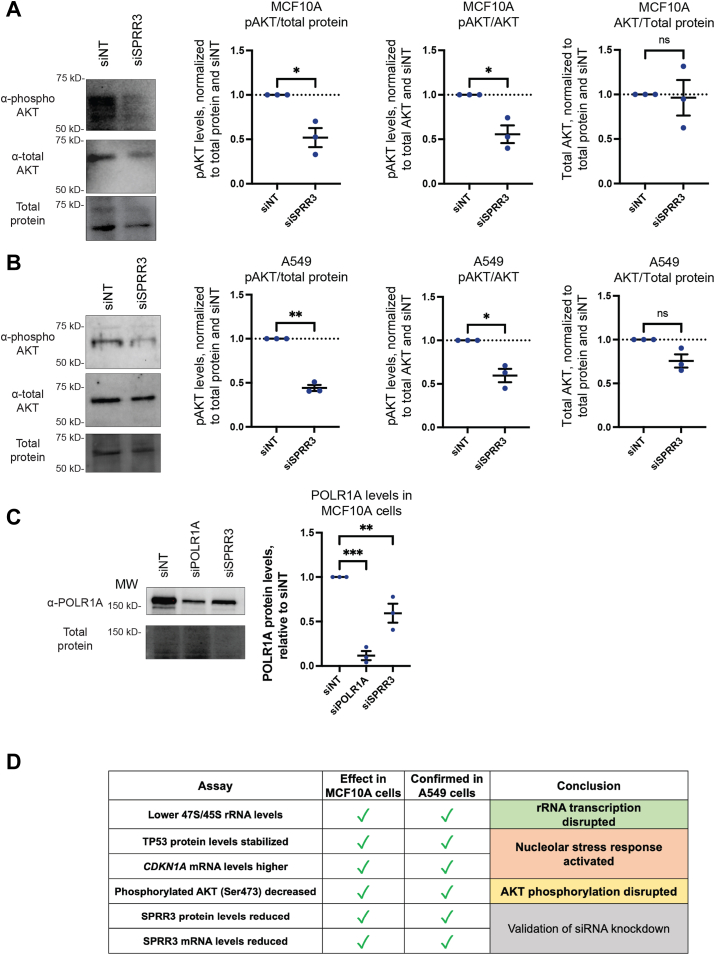
**SPRR3 drives AKT phosphorylation and maintains POLR1A levels.***A*, AKT phosphorylation at serine 473 (pAKT) is decreased after a 72h SPRR3 depletion in MCF10A cells. Representative images and quantification are shown for pAKT, total AKT, and total protein (measured by trichloroethanol stain). Blots were probed for pAKT, then stripped and re-probed for total AKT. Signal was measured in Bio-Rad Image Lab. pAKT levels were normalized to total protein and total AKT. Total AKT was normalized to total protein, ensuring no significant difference in overall AKT levels upon SPRR3 depletion. *B*, phosphorylated AKT (pAKT) levels are decreased after 72h SPRR3 depletion in A549 cells. Representative images and quantification are shown for pAKT, total AKT, and total protein (measured by trichloroethanol stain). *C*, POLR1A levels are decreased upon 72h SPRR3 depletion. Representative image of Western blotting and quantification of POLR1A levels in MCF10A cells. siPOLR1A was used as a positive control. *D*, summary of effects of siSPRR3 depletion that we have confirmed in both MCF10A cells and A549 cells. For all graphs in this figure, the mean ± SEM are shown alongside individual data points. Data were analyzed by unpaired two-sided Welch's *t*-tests in GraphPad Prism. ∗, *p* < 0.05; ∗∗, *p* < 0.01; ∗∗∗, *p* < 0.001; ns, not significant.

To determine whether pAKT levels were reduced upon depletion of SPRR3, we performed Western blotting after a 72 h depletion in both MCF10A cells and A549 cells. The level of pAKT was measured by pAKT/total AKT. The pAKT/total protein and AKT/total protein ratios were also determined to establish if SPRR3 influences total AKT production and to test for an overall decrease in pAKT levels, an analysis based on previous work studying pAKT reduction after SPRR3 depletion (19). In MCF10A cells, the pAKT/AKT and pAKT/total protein ratios after siSPRR3 treatment were significantly decreased to about 50% compared to the siNT negative control, providing evidence for reduced AKT phosphorylation (Fig. 5*A*). In contrast, siSPRR3 did not reduce total AKT levels in MCF10A cells, as shown by the AKT/total protein ratio (Fig. 5*A*). Similar to the results in MCF10A cells, the pAKT/AKT and pAKT/total protein ratios in A549 cells were significantly reduced to around 50%, while total AKT levels were unaffected (Fig. 5*B*). We conclude that in both cell lines, SPRR3 depletion reduces the phosphorylation of AKT, as observed in previous reports (18, 19).

Because depletion of SPRR3 resulted in reduced pre-rRNA transcription, we asked whether the levels of the essential RNAPI subunit POLR1A were also reduced, previously described as a mechanism by which non-nucleolar proteins reduce pre-rRNA transcription (29). To test this, we measured the levels of POLR1A, the catalytic subunit of RNAPI, by Western blot after 72 h of SPRR3 depletion. siPOLR1A was used as a positive control, and reduced levels of that protein to 12%. Upon siSPRR3 treatment, POLR1A levels were reduced to 60% of siNT-negative control values, consistent with the conclusion that SPRR3 plays a role in maintaining RNAPI levels under normal conditions (Fig. 5*C*).

In sum, in addition to our initial screening experiments and biochemical assays to probe the role of SPRR3 in ribosome biogenesis in MCF10A cells, we have confirmed in both A549 and MCF10A cells that SPRR3 depletion reduces levels of the 47S/45S rRNA, stabilizes TP53 levels, increases *CDKN1A* mRNA levels, and decreases AKT phosphorylation (summarized in Fig. 5*D*). This predicts a mechanism in which SPRR3 depletion reduces AKT phosphorylation, resulting in lower levels of an RNAPI subunit and thus transcription of rRNA, subsequently triggering the nucleolar stress response.

## Discussion

In this work, we identify SPRR3 as a regulator of pre-rRNA transcription in the nucleolus. We initially uncovered this relationship between SPRR3 and ribosome biogenesis *via* a high-throughput screen for nucleolar number. We show in MCF10A breast epithelial and A549 lung carcinoma cell lines that SPRR3 depletion reduces pre-rRNA transcription as measured by RT-qPCR. We confirm *via* an rDNA promoter activity reporter assay and 5-EU imaging that pre-rRNA transcription depends on normal SPRR3 levels in MCF10A cells. We show that loss of SPRR3 leads to increased TP53 protein and *CDKN1A* mRNA levels in both cell lines studied, emblematic of the nucleolar stress response. Finally, we confirm that loss of SPRR3 reduces AKT phosphorylation and report that levels of the essential RNAPI subunit POLR1A are decreased upon SPRR3 depletion. As SPRR3 is a non-nucleolar protein, we posit that SPRR3 mediates its role in pre-rRNA transcription through its previously reported regulation of AKT phosphorylation (18, 19). This previously undescribed relationship between SPRR3 and ribosome biogenesis represents an important contribution to understanding the mechanisms by which SPRR3 regulates proliferation and tumorigenesis.

While SPRR3 was initially discovered as a potential tumor suppressor in esophageal cancer (21, 22, 45, 46), it has emerged as an oncogenic driver of cell proliferation and tumorigenesis in several other cancer types, including renal, colorectal, pancreatic, non-small cell lung, breast, and glioblastoma multiforme (17, 18, 19, 20, 23, 47, 48, 49). Why SPRR3 has proliferative effects in these cancers, but a suppressive effect in esophageal cancer, remains unknown. The mechanism by which SPRR3 drives proliferation in these cases also remains to be fully described. Interestingly, previous work suggested that TP53 stabilization may play a role in SPRR3’s proliferative effects, as cell lines stably expressing (17) or overexpressing SPRR3 (47) have lower TP53 levels, possibly related to increased phosphorylation of MDM2 (17, 47). Our work likewise supports that SPRR3 expression is inversely correlated with TP53 levels. We propose that this occurs through the well-studied nucleolar stress response triggered by inhibition of ribosome biogenesis.

Taken together, our results synthesize two existing mechanisms related to cell proliferation and cancer: increased AKT phosphorylation by SPRR3 (18, 19) and activation of RNAPI by AKT phosphorylation (25, 26). We demonstrate that transcription of rRNA is mediated by SPRR3, which agrees with existing literature on the relationship between RNAPI and AKT (25, 26). We confirm that SPRR3 facilitates AKT phosphorylation and propose that it therefore functionally leads to RNAPI activity in pre-rRNA transcription. Previous work has shown that RNAPI elongation and loading are diminished upon inhibition of AKT phosphorylation, and that AKT phosphorylation promotes pre-rRNA transcription by enhancing RRN3 (aka TIF-1A) stabilization in the RNAPI transcription initiation complex (25, 26). We observed lower levels of the RNAPI subunit POLR1A upon SPRR3 depletion. It is possible that this POLR1A decrease is downstream of a destabilization of the RNAPI transcription machinery, caused by the reduced RNAPI loading and decreased RRN3 stabilization described in previous work (25, 26). Interestingly, the specific RNAPI inhibitor BMH-21 has been shown to cause proteasome-mediated degradation of POLR1A upon inhibition of pre-rRNA synthesis (33, 34, 50), so it is plausible that a similar degradation occurs after the inhibition of rRNA synthesis that we observe after SPRR3 depletion. Additionally, we have shown a role in cell growth and stress for SPRR3, demonstrating that its loss activates the nucleolar stress response, which coordinates the process of ribosome biogenesis with cell proliferation and death (51).

We can speculate here on the ways that SPRR3 may regulate levels of AKT phosphorylation. The mTORC2 complex carries out AKT phosphorylation at serine 473 and threonine 308 (52). SPRR3 has been shown to interact with AKT in high-throughput proteomics (53, 54) and may directly bind it to facilitate normal levels of mTORC2-mediated AKT phosphorylation. SPRR3 has been reported to localize to the cytoplasm (11, 55) (https://www.uniprot.org/uniprotkb/Q9UBC9/entry, Accessed December 11, 2025; https://www.ptglab.com/products/SPRR3-Antibody-11742-1-AP.htm, Accessed December 10, 2025); cell periphery (11, 56); mitochondria (57); Golgi apparatus (part of the endomembrane system) (58); and perinucleus (55) but is absent from the nucleus (56, 58). mTORC2 phosphorylation of AKT has been shown to occur at the plasma membrane (52, 59), endomembrane system (52, 60), mitochondria-associated ER (61), and mitochondria (59). SPRR3 subcellular localization thus overlaps with known sites of mTORC2 activity on AKT (endomembrane system, mitochondria, plasma membrane), supporting a role for SPRR3 in modulating mTORC2’s kinase activity.

Our findings have important implications for the treatment of cancers with increased SPRR3 expression, as they suggest that its oncogenicity is driven by upregulation of ribosome biogenesis. Increased ribosome biogenesis and nucleolar dysmorphology have long been associated with cancer (38, 62, 63, 64, 65). RNAPI inhibition and nucleolar stress are mechanisms underlying existing cancer drugs (39, 66, 67, 68, 69, 70), and targeted RNAPI inhibitors are being explored as novel cancer treatments (33, 34, 50). Due to its multifaceted role in regulating proliferation, AKT has also been studied as a target for chemotherapy (71, 72, 73), and its dysregulation has been suggested as a mechanism of chemotherapy resistance (72, 74). Our results suggest that overexpression of SPRR3 drives the excessive proliferation of cancer cells by regulating pre-rRNA transcription, mediated by AKT phosphorylation. In this model, both AKT phosphorylation and RNAPI activity appear as potential targets for cancer treatments in cases where SPRR3 is a driver of tumorigenesis.

In all, we have shown that SPRR3 acts as an activator of ribosome biogenesis outside of the nucleolus and upstream of direct rDNA transcription, mediated by the phosphorylation of AKT. This function is consistent with the reported cytoplasmic localization of SPRR3 in previous work (75) and agrees with SPRR3’s known oncogenic function in several cancer types (17, 19, 20, 47, 48, 49). Our results elucidate the regulatory influence of this small signaling protein at the nexus of cytoplasmic and nucleolar processes, underscoring the importance of the non-nucleolar regulation of ribosome biogenesis.

## Experimental procedures

### Screening data analysis

The complete methods for the siGENOME screen can be found in (6). Normalized percent effect (one nucleolus) values were used to rank the siRNAs, and screening statistics were robust (6). The SPRR3 depletion resulted in a percent effect >2 standard deviations above the mean normalized values for the entire screening population.

### Cell culture

Human MCF10A breast epithelial cells (#CRL-10317, American Type Culture Collection) were cultured in DMEM/nutrient mixture F-12 (Gibco 11330032) with 5% horse serum (Gibco 16050122), 20 ng/ml epidermal growth factor (Peprotech AF1005), 0.5 μg/ml hydrocortisone (MilliporeSigma H0135), 100 ng/ml cholera toxin (MilliporeSigma C8052), and 10 μg/ml insulin (MilliporeSigma I1882). A549 human lung carcinoma cells (#CCL-185, American Type Culture Collection) were cultured in DMEM with 10% Fetal Bovine Serum (FBS) and 1% Penicillin-Streptomycin. All cells were incubated in a humidified atmosphere with 5% CO_2_ at 37 °C.

### siRNA and BMH-21 treatment

Cells were seeded at 100,000 cells/well in 2 ml of media in 6-well plates and incubated for 24 h at 37 °C. Cells were transfected with siRNAs using Opti-MEM (Gibco 31985070) and Lipofectamine RNAiMAX (Invitrogen 13778-150) per manufacturer’s instructions to a total volume of 2250 μl. Except when noted, for the siSPRR3 treatment, cells were transfected with the custom subpool of 30 nM siSPRR3-si1 and siSPRR3-si2. Cells were transfected with 30 nM siRNAs for control treatments. siRNA sequences and product numbers can be found in Table S1. BMH-21 (Sigma-Aldrich SML1183; CAS 896705-16-1) was added 24 h before assays were performed, to a final concentration of 1 μM.

### 5-Ethynyl uridine labeling, staining, and high-content imaging

5-ethynyl uridine (5-EU; ClickChemistryTools 1261-100, CAS 69075-42-9) was used to label cells at a 1 mM final concentration. Complete methods for staining, click chemistry, high-throughput imaging, and quantification are described in (30).

### RNA isolation and analysis of RNA transcript levels by RT-qPCR

Cells were washed with 1X PBS, then collected with 1 ml of TRIzol reagent (Invitrogen 15596026). Total RNA was purified following the manufacturer’s protocol. cDNA was synthesized from 1 μg total input RNA using iScript gDNA Clear cDNA Synthesis Kit (Bio-Rad 1725035). In a qPCR plate (Bio-Rad MLL9601), 1 μl of cDNA was mixed with 19 μl of a qPCR master mix containing iTaq Universal SYBR Green Supermix (Bio-Rad 1725121), 500 nM F primer, 500 nM R primer, and water. Primer sequences can be found in Table S2. The plate was briefly centrifuged, then assayed using a Bio-Rad CFX96 Touch Real-Time PCR Detection System. Amplification parameters were as follows: initial denaturation 95 °C for 30 s; 40 cycles 95 °C denaturation for 15 s, 60 °C annealing and extension for 30 s, except for *SPRR3* mRNA detection in A549 cells, which was for 60 cycles. The primers used to detect *SPRR3* mRNA levels in MCF10A cells were specific to the *SPRR3-v2* transcript (46). For A549 cells, we used primers that could detect both known splicing variants and had previously been used for published work in A549 cells (18). The Ct values to detect the *SPRR3* transcript in MCF10A cells were around 24 in negative control samples, and around 40 for the same conditions in A549 cells. Melt curve analysis parameters were: 60 °C to 94.8 °C in 0.3 °C increments. Data analysis was completed using the comparative C_T_ method (ΔΔC_T_) using 7SL RNA as an internal loading control.

### Analysis of mature rRNAs

RNA from treated MCF10A cells was isolated as described above in the section “RNA isolation and analysis of RNA transcript levels by RT-qPCR.” Total RNA was resuspended in nuclease-free H_2_O and 1 μg of RNA was submitted to the Yale Center for Genomic Analysis for electropherogram analysis. The experiment was conducted with a Fragment Analyzer 5300 (Agilent). Mature rRNA ratios and mature rRNA relative peak areas were taken from the output reports. The data were graphed and analyzed by ANOVA followed by Holm-Šídák *post hoc* testing in GraphPad Prism.

### Northern blot analysis of pre-rRNA processing

MCF10A cells were treated with the indicated siRNAs, and RNA was isolated as above. Northern blots were performed using 3 μg of total RNA as published (6, 7) and were performed in at least biological triplicate. Blots were quantified with Image Lab 6.0.1 (Bio-Rad). RAMP (Ratio Analysis of Multiple Precursors) (76) ratios were calculated in Microsoft Excel, and heatmaps were made using the mean log_2_ RAMP ratio for each treatment relative to siNT in GraphPad Prism. The following DNA oligonucleotide was radiolabeled for blotting as the P3 probe/ITS1 probe: (5′ → 3′), AAGGGGTCTTTAAACCTCCGCGCCGGAACGCGCTAGGTAC (6, 32).

### Dual-luciferase assay for rDNA promoter activity

MCF10A cells were seeded at 30,000 cells per well in 1 ml of media in 12-well plates and incubated at 37 °C for 24 h. Th next day, 72 h before harvesting, cells were transfected with 30 nM siRNAs as above. 24h before harvest, cells were transfected with 1 μg of pHrD-IRES-Fluc and 1 ng of CMV-Rluc reporter plasmids (10) using Lipofectamine 3000 (Invitrogen L3000015). Also 24 h before harvest, cells were treated with 3.5 μl of DMSO vehicle or 300 μM BMH-21, to achieve a final concentration of 1 μM BMH-21. To harvest, cells were washed once with 1X PBS and lysed with 250 μl of 1X Passive Lysis Buffer (Promega E1941) at RT for at least 30 min. In a solid white 96-well plate (Greiner Bio-One 655074), 20 μl of lysate from each sample was dispensed into a well. Samples were assayed using a Promega GloMax plate reader with dual injectors, using the Dual-Luciferase Reporter Assay System (Promega E1910) per manufacturer’s instructions. 60 μl of LAR II or Stop & Glo substrate were injected with a 2 s delay and a 10 s read time. Data were analyzed by calculating the Fluc/Rluc ratio for each well, then normalizing to the Fluc/Rluc ratio for siNT. Data import was carried out in Microsoft Excel, calculations were performed in JMP, and normalized data were graphed and analyzed by ANOVA followed by Holm-Šídák *post hoc* testing in GraphPad Prism.

### Puromycin incorporation translation assay

After siRNA transfection, MCF10A cells were treated with a final concentration of 1 μM (0.5 μg/ml) puromycin (Mirus Bio 5940) and final volume of 3 ml. Cells were incubated 1 h at 37 °C, then washed with cold 1X PBS. Protein was isolated and analyzed by immunoblotting as described below.

### Protein isolation, SDS-PAGE analysis, and immunoblotting

For all protein preparations other than pAKT/AKT Western blots: after siRNA treatment, cells were washed twice with 1X cold DPBS (Sigma-Aldrich D8537), manually dislodged with cell scrapers (Falcon 355085), collected in 1.2 ml 1X DPBS, and pelleted by centrifugation at 1100 rcf at 4 °C for 5 min, then lysed in AZ lysis buffer (50 mM Tris pH 7.5, 250 mM NaCl, 1% Igepal, 0.1% SDS, 5 mM EDTA pH 8.0) with 1X complete protease inhibitors (cOmplete Protease Inhibitor Cocktail, Roche 11697498001) by vortexing for 15 min in a cold room. Cell debris was removed by pelleting at 21,000 rcf for 15 min. The concentration of total protein in the supernatant was determined by Bradford assay, and protein aliquots were prepared with 5X Laemmli buffer, boiled at 95 °C for 5 min, and loaded onto a gel or frozen at −20 °C until use.

For pAKT/AKT protein preparations, cells were washed with 1X PBS and pelleted by centrifugation at 18,000 rcf at 4 °C for 2 min. Cell pellets were dissolved directly into 2X protein loading dye (PLD) (75 mM Tris-HCl, pH 6.8, 1.25 mM EDTA pH 8, 20% glycerol, 2.5% SDS, 0.125% bromophenol blue, 62.5 mM DTT) until homogenized and heated at 95 °C for 5 min before loading.

Handcast SDS-PAGE stacking gels of appropriate percentage for the protein of interest were run until the dye front reached the bottom of the gel. The gels contained 0.5% (v/v) trichloroethanol (Acros Organics 139441000) to allow use of the ChemiDoc stain-free imaging protocol (Bio-Rad) for verification of even protein loading at the gel stage and normalization of total protein after protein transfer. Gels were UV-activated according to Bio-Rad protocol and imaged in the ChemiDoc then placed in the Bio-Rad Trans-Blot Turbo system for membrane transfer according to manufacturer’s instructions. Following the transfer, blots were imaged again for total protein; these images are presented and quantified as loading controls.

Immunoblotting was carried out using 5% (w/v) dry milk in 1X PBST with primary antibodies listed in Table S3 (with references), followed by 1:5000 peroxidase-linked anti-mouse or anti-rabbit IgG (Amersham NXA931 or NA934) as appropriate. Blots were developed using high-sensitivity ECL reagent (Thermo Scientific 34094) for 2 to 5 min, then imaged with the ChemiDoc. Images were quantified using Bio-Rad Image Lab per manufacturer’s instructions. Data were graphed and analyzed by ANOVA followed by Holm-Šídák *post hoc* testing in GraphPad Prism.

To strip blots, 10 μl of BME was added to 10 ml 1X stock stripping buffer (62.5 mM Tris pH 6.8, 2% SDS) just prior to use. The membrane was put into a hybridization tube with the stripping buffer and incubated in the hybridization oven at 65 °C for 20 min. Membrane was washed with PBST (2X quick washes, 2X 5-min washes), then tested with ECL reagent to ensure no residual antibody remained before rinsing again with PBST, re-blocking and probing with the next antibody.

## Data availability

All data supporting the findings of this study can be found in this document. Full information on the nucleolar number screen can be found in (6), and data supporting the 5-EU assay can be found in (30). Other information such as raw data is available from the corresponding author upon request.

## Supporting information

This article contains supporting information.

## Conflict of interest

The authors declare that they do not have any conflicts of interest with the content of this article.

## Supporting information

Supporting informtion
