# Supplementary material for: A high-throughput screen for nucleolar function reveals a role for the signaling protein, SPRR3, in ribosome biogenesis
Source: J Biol Chem. 2026 Jan 7;302(3):111132. doi: 10.1016/j.jbc.2026.111132 (PMC12876706; doi:10.1016/j.jbc.2026.111132)
Supplement: Supporting informtion [file mmc1.pdf]

Supporting Information

A high throughput screen for nucleolar function reveals a role for the signaling protein, SPRR3, in ribosome biogenesis

Emily C. Sutton*, Carson J. Bryant*, Janina I.S. Gbenoba, Isabella R. Lawrence, and Susan J. Baserga

*These authors contributed equally to this work

Correspondence: susan.baserga@yale.edu

List of Supporting Information:

Figure S1. Bioanalyzer analysis of total RNA from MCF10A cells after SPRR3 depletion shows no changes to 28S/18S rRNA ratio.

Figure S2. Northern blots reveal no changes in pre-rRNA processing after SPRR3 depletion in MCF10A cells

Table S1. siRNA used in this study

Table S2. RT-qPCR primer sequences

Table S3. Primary antibodies used in this study

Figure S1

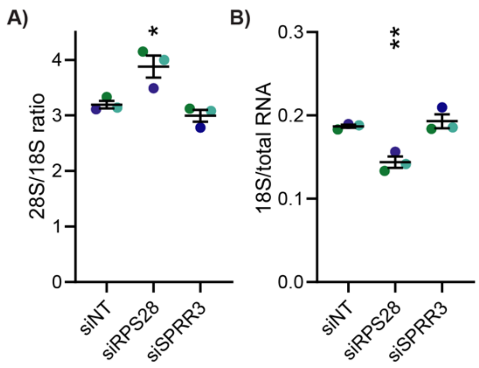

Figure S1. Bioanalyzer analysis of total RNA from MCF10A cells after SPRR3 depletion shows no changes to 28S/18S rRNA ratio.

Bioanalyzer analysis for 1 µg of total RNA isolated from SPRR3-depleted MCF10A cells. A) The 28S/18S mature rRNA ratio. B) the 18S mature rRNA/total RNA ratio. Mean ± SEM are shown alongside individual data points, colored by replicate. The data were graphed and analyzed by ordinary one-way ANOVA with multiple comparisons against a non-targeting siRNA (siNT, negative control) and Holm-Šídák correction in GraphPad Prism. *, $p < 0.05$; **, $p < 0.01$. The negative control and siRPS28 data have been published in (1), while the siSPRR3 condition was not previously published. They are included herein to demonstrate that the negative and positive (siRPS28) controls were as expected.

Figure S2

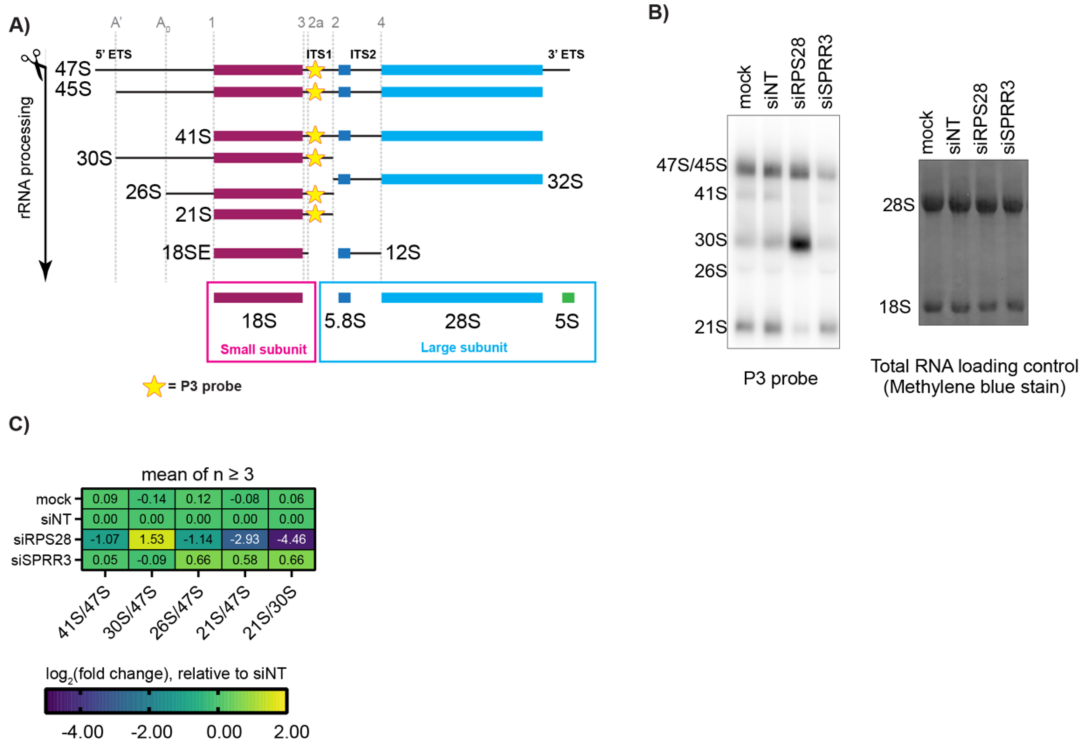

Figure S2. Northern blots reveal no changes in pre-rRNA processing after SPRR3 depletion in MCF10A cells

A) Schematic of ribosomal RNA processing indicating location of the ITS1 (P3) probe. B) Representative ITS1 (probe P3) northern blot of 3 µg of total RNA isolated from SPRR3-depleted MCF10A cells, with pre-rRNA processing intermediates. Mock is the no siRNA transfection control. siNT is non-targeting siRNA negative control. siRPS28 is included to show example of pre-rRNA processing defects. Methylene blue staining for total protein of the blot is shown on the right, demonstrating even loading. C) A heat map showing log2-transformed Ratio Analysis of Multiple Precursor (2) calculations, normalized to the siNT negative control. The values represent mean RAMP ratio for n = 4 replicates, except n = 3 for mock. The images were quantified using Bio-Rad Image Lab. RAMP ratios were calculated in Microsoft Excel and data

were graphed in GraphPad Prism. The negative control and siRPS28 results have been published in (1), while the siSPRR3 condition was not previously published.

Supplemental Tables

Table S1: siRNA used in this study

siRNA	Product number (all from Horizon Discovery)	Sequence(s)
siSPRR3-si1	J-019976-09	CAGCAGAAGCAGACCUUUA
siSPRR3-si2	J-019976-10	CCAUCAAAGUUCCUGAGCA
siSBDS (SMARTpool)	L-019217, pool containing:	
	J-019217-05	UUAGAAAUCGUAUGUCUGA
	J-019217-06	GUAAGCAGAUUUUGACUAA
	J-019217-07	UCAAGGUCAUAGAAAGUGA
	J-019217-08	GAGAUGAGAAAUUUGAAUG
siRPL4 (SMARTpool)	L-008956, pool containing:	
	J-008956-09	GAAGAAGCCUGCUGCAUAA
	J-008956-10	CUAUGCUGUCAGUGAAUUA
	J-008956-11	AGUGCUGAGUCUUGGGGUA
	J-008956-12	CAGCGGCACUACAAGCCAA
siNOL11 (SMARTpool)	L-016695, pool containing:	
	J-016695-09	UACCUGAAGUGUAGCGAAA
	J-016695-10	CAUUCAGCAUAUAGCGAGA
	J-016695-11	UGAAGUAGAAGUACGGAAA
	J-016695-12	CGUCGCAGUCCUAGGAAGU
ON-TARGETplus Non-targeting Pool (siNT)	D-001810-10	UGGUUUACAUGUCGACUAA
		UGGUUUACAUGUUGUGUGA
		UGGUUUACAUGUUUUCUGA
		UGGUUUACAUGUUUUCCUA
siPOLR1A	L-013983, pool containing:	
	J-013983-09	GCAAACGGCCUGUACGAUU
	J-013983-10	CAUCAACACCAACGAAAUU
	J-013983-11	ACAUGCAGGAGGAACGAAA
	J-013983-12	ACUAAUGGCCAGACGGUGA

Table S2: RT-qPCR primer sequences

**Target RNA**	**Forward primer (5' → 3')**	**Reverse primer (5' → 3')**	**Ref.**
47S/45S pre-rRNA	GAACGGTGGTGTGTCGTTC	CGTCTCGTCTCGTCTCACTC	(3)
7SL RNA	ATCGGGTGTCCGCACTAAGTT	CAGCACGGGAGTTTTGACCT	(4)
CDKN1A (AKA *p21*)	TGGAGACTCTCAGGGTCGAAA	GGCGTTTGGAGTGGTAGAAATC	(5)
SPRR3-v2 (MCF10A)	AGCAGGTCCAGCATCCTTTGA	CTCCTTGGTTGTGGGAACAAAT	(6)
SPRR3 (A549)	ATGAGTTCTTACCAGCAGAAGC	GTTCAGGGACCTTGGTGTAGC	(7)

Table S3: Primary antibodies used in this study

**Target molecule**	**Primary antibody manufacturer and catalog number**	**Dilution**
FBL	72B9 (from hybridoma grown in house; heavy and light chain plasmids available on AddGene, ID 194637 and 194638) (8)	1:1000
POLR1A	Santa Cruz Biotechnology sc-48385 (clone C-1)	1:1000
Puromycin	Kerafast EQ0001 (clone 3RH11)	1:5000
SPRR3	Proteintech 11742-1-AP	1:500
TP53	Santa Cruz Biotechnology sc-126 HRP (clone DO-1)	1:5000
Phospho-AKT (Ser473)	Cell Signaling Technology (#4060S)	1:1000
AKT	Proteintech 10176-2-AP	1:1000

References for supporting information

1. Bryant, C. J., McCool, M. A., Rosado González, G. T., Abriola, L., Surovtseva, Y. V., and Baserga, S. J. (2024) Discovery of novel microRNA mimic repressors of ribosome biogenesis. *Nucleic Acids Research*. 10.1093/nar/gkad1235
2. Wang, M., Anikin, L., and Pestov, D. G. (2014) Two orthogonal cleavages separate subunit RNAs in mouse ribosome biogenesis. *Nucleic Acids Res*. **42**, 11180–11191
3. Woolnough, J. L., Atwood, B. L., Liu, Z., Zhao, R., and Giles, K. E. (2016) The Regulation of rRNA Gene Transcription during Directed Differentiation of Human Embryonic Stem Cells. *PLoS One*. **11**, e0157276
4. Galiveti, C. R., Rozhdestvensky, T. S., Brosius, J., Lehrach, H., and Konthur, Z. (2010) Application of housekeeping npcRNAs for quantitative expression analysis of human transcriptome by real-time PCR. *RNA*. **16**, 450–461
5. Hanashima, Y., Sano, E., Sumi, K., Ozawa, Y., Yagi, C., Tatsuoka, J., Yoshimura, S., Yamamuro, S., Ueda, T., Nakayama, T., Hara, H., and Yoshino, A. (2020) Antitumor effect of lenalidomide in malignant glioma cell lines. *Oncol Rep*. **43**, 1580–1590
6. de A Simão, T., Souza-Santos, P. T., de Oliveira, D. S. L., Bernardo, V., Lima, S. C. S., Rapozo, D. C. M., Kruel, C. D. P., Faria, P. A., Ribeiro Pinto, L. F., and Albano, R. M. (2011)

Quantitative evaluation of SPRR3 expression in esophageal squamous cell carcinoma by qPCR and its potential use as a biomarker. *Exp Mol Pathol*. **91**, 584–589

7. Li, Q., Wang, Y., Hu, R., and Yang, G. (2020) Dysregulation of SPRR3/miR-876-3p Axis Contributes to Tumorigenesis in Non-Small-Cell Lung Cancer. *Onco Targets Ther*. **13**, 2411–2419
8. Reimer, G., Raška, I., Tan, E. M., and Scheer, U. (1987) Human autoantibodies: probes for nucleolus structure and function. *Virchows Archiv B Cell Pathol*. **54**, 131–143
